# Management of Αcute Non-Variceal Upper Gastrointestinal Bleeding: Drugs, Endoscopic Hemostasis, or Both?

**DOI:** 10.4021/gr2008.12.1253

**Published:** 2009-01-20

**Authors:** Stelios F. Assimakopoulos, Konstantinos C. Thomopoulos

**Affiliations:** a Department of Internal Medicine, University Hospital of Patras, Patras 26504, Greece; b Division of Gastroenterology, Department of Internal Medicine, University Hospital of Patras, Patras 26504, Greece

## Abstract

Acute upper gastrointestinal bleeding remains one of the most frequent and emergent conditions in everyday clinical practice and a challenge for doctors. Peptic ulcer is responsible for more than half of acute upper gastrointestinal bleeding and is the most frequent cause of serious non-variceal bleeding. Despite progress in diagnosis and management in these patients, the recurrence of bleeding remains an important problem. Several drugs and endoscopic techniques, alone or in combination, have been evaluated in many studies and there is presently enough experience in terms of their efficacy. Endoscopic hemostasis is more effective than any other therapeutic intervention in the treatment of patients with non-variceal upper gastrointestinal bleeding. In patients with high risk of rebleeding spots, the combination of endoscopic hemostasis with high dose proton pump inhibitors is the most effective strategy to reduce bleeding recurrences and the need for surgery.

## Introduction

Acute upper gastrointestinal bleeding continues to be one of the most frequent and emergent conditions in everyday clinical practice and a challenge for doctors, despite progress in diagnosis and management in these patients. Variceal rupture accounts for 6%-30% of cases, while in other cases, diseases related to the deleterious effects of hydrochloric acid on gastro-duodenal mucosa are the cause of the bleeding [1, 2]. Peptic ulcer is responsible for more than half of acute upper gastrointestinal bleeding and is the most frequent cause of severe non-variceal bleeding, with duodenal ulcer being far more frequent as compared to stomach ulcer [1, 3]. In recent years, the improved management of patients with chronic duodenal ulcers (eradication of helicobacter pylori) has led to a reduction in bleeding from idiopathic duodenal ulcers [4, 5]. On the other side, an increase in the incidence of bleeding from ulcers related to non steroidal anti-inflammatory and antiplatelet drugs has been observed affecting typically elderly population [6].

Severity of bleeding on admission varies widely, from non significant to catastrophic. Eighty percent of bleeding cases stops spontaneously; while 20% of patients continue to bleed or rebleed, this aggravates morbidity and increases the need for emergent surgical hemostasis and mortality [1, 3, 7]. The overall mortality of acute upper gastrointestinal bleeding ranges is from 8 to 14%, it is typically higher in inpatient group and older patients, and is mainly attributed to coexisting diseases, which are more frequent in older patients, rather than to oligaemic shock from blood loss [1, 6, 8].

## Therapeutic interventions in patients with acute upper non variceal bleeding

Despite advances, emergency surgical haemostasis is the only choice for the patient with ongoing life-threatening non-variceal upper gastrointestinal bleeding so far. The increase in the average age of patients and the increased prevalence of coexisting diseases, particularly the cardiovascular diseases, in hospitalised patients with bleeding gave impetus for the design and study of a large number of non-surgical therapeutic interventions, such as pharmaceutical and/or endoscopic. The aim was to achieve hemostasis of the bleeding vessels and to prevent rebleeding using less interventional means, thus to improve clinical outcome and reduce mortality in these patients. The ideal therapy would be one that would both facilitate hemostasis and prevent the dissolution of the clot. The non-surgical therapeutic interventions include drugs, which support directly or indirectly the clot formation and stabilization, and endoscopic hemostasis.

The drugs which have been used in acute non-variceal bleeding and in particular peptic ulcer bleeding affect the natural history of bleeding in three ways. (a) reducing hydrochloric acid secretion and thus creating a more favourable environment for the healing of the lesion and clot stabilization; (b) reducing or delaying clot dissolution;(c) reducing splachnic blood flow.

Several drugs and endoscopic techniques alone or in combination have been used in many studies and there is now enough experience in terms of their effectiveness.

## Pharmaceutical treatment

### Somatostatin – Octreotide

Although originally proposed for the treatment of patients with non-variceal bleeding, on the ground that they can reduce both splachnic blood flow and gastric acid secretion, there is no clear evidence that these drugs have any beneficial effect in the treatment of patients with non-variceal bleeding and are not routinely indicated [9]. However, in a subgroup of patients who are bleeding uncontrollably while awaiting endoscopy or in patients with non-variceal bleeding who are awaiting surgery or for whom surgery is contraindicated, this therapy might be useful in light of the favourable safety profile of these medications in the acute setting [9].

### Histamine H_2_-receptor antagonists

Histamine H_2_-receptor antagonists are weak suppressants of hydrochloric acid secretion even when given in high doses continuously intravenous. An initial 1985 meta-analysis by Collins and Langman, which included 27 randomized trials with more than 2500 patients, suggested that H_2_-receptor antagonist treatment might reduce the rates of rebleeding, surgery, and death by approximately 10%, 20%, and 30%, respectively, compared with placebo or usual care [10]. However, more recent meta analyses have demonstrated that these drugs are significantly less effective than proton pump inhibitors and their mild efficacy is confined in patients with bleeding gastric ulcer, whilst are of no value in bleeding duodenal ulcers [11, 12]. Given the proven benefit of proton-pump inhibitors and the inconsistent and at best marginal benefits of H_2_-receptor antagonists, the latter are not recommended for the management of acute upper GI bleeding [9].

### Proton pump inhibitors

Proton pump inhibitors are powerful inhibitors of hydrochloric acid secretion achieving higher levels of gastric pH. Previous studies have shown that gastric pH above 6 promotes aggregation of platelets, inhibits activation of pepsin and prevents clot dissolution [13-15]. The levels of gastric pH achieved are not different in oral or intravenous administration. The administration of 40 mg pantoprazole orally or intravenously achieved the same profile of hydrochloric acid supression in healthy volunteers, although at this dosage suppression was not complete at least the first day of administration [16]. The question is whether higher doses and intravenous administration of proton pump inhibitors can achieve earlier, more intense and stable suppression of gastric acid secretion and therefore more favourable environment for the stabilization of the clot, and whether this translates into lower rates of clinical rebleeding.

It is known that more frequent doses and especially continuous administration of proton pump inhibitors at a dose of 8 mg/hr after a loading dose of 80mg achieves greater suppression of hydrochloric acid secretion and higher intragastric pH even from the first day of treatment [17]. In recent studies, the continuous high dose proton pump inhibitor administration achieved levels of gastric pH above 6 for a longer period than the standard dose, but even with this regimen the duration that pH was above 6 ranged from 27.7% to 84% of the 24 hour period [18]. The difference is attributable to genetic factors (drug metabolism) and/or the presence of gastric atrophy from infection by Helicobacter pylori (developed vs. developing countries). In most cases, high doses of proton pump inhibitors continuously intravenous achieve significant suppression of gastric acid secretion directly even in the first 24 hours. It is worth noting that high doses are well tolerated by patients without creating adverse effects [18, 19].

In a recent study, the importance of pH achieved after intravenous administration of high dose omeprazole was investigated and was inversely associated with the recurrence of bleeding [20]. The recurrence of bleeding within 72 hours was significantly higher in patients with medium _P_H less than 6 in the first 24 hours (4.8% vs. 20%, p = 0.03).

Several studies have tested the value of administration of proton pump inhibitors either by mouth or intravenously to patients with non-variceal upper gastrointestinal bleeding compared with placebo (Table 1) [21-24]. Significant improvement in clinical outcome of patients by the administration proton pump inhibitors orally without any endoscopic intervention was observed in a study from Asia [25]. The administration of omeprazole 40 mg x 2 orally for 5 days reduced the rebleeding (10.9% vs. 36%) and the need for emergent surgical hemostasis (7.3% vs. 23.6%) without affecting mortality compared with placebo.

**Table 1:** Effect of PPIs at diverse doses in the clinical outcome of patients with bleeding peptic ulcers

Studies (Ref. No)		N	Rebleeding	Surgery	Mortality
**Daneshmend et al. 1992 **[21] 1147 patients with AUGIB (45% Peptic ulcer, 18% erosions)	Omeprazole 80mg followed by 40mg x 3 IV	578	18%	11%	5.3%
	Placebo	569	1 5%	11%	6.9%
	Comments: Reduction of stigmata of active or recent bleeding at endoscopy				
**Kurhoo et al. 1997 **[22] 220 patients with peptic ulcer and stigmata of active or recent bleeding. (exclusion of 9 massive bleeding cases)	Omeprazole 40mg x 2 orally	110	10.9%	7.3%	1.8%
	Placebo	110	40%*	23.6%*	5.4%
	Comments: Younger patients as compared with the western world patients suffering from AUGIB				
**Hasselgren et al. 1997 **[23] 322 patients ≥60 years old with AUGIB	Omeprazole 80mg bolus followed by 8mg/h	159	10%	8.1%	6.9%*
	Placebo	163	18.5%*	14.1%*	0.6%
**Βarkun et al. 2004 **[24] 1244 patients >60 years old with bleeding peptic ulcer	Pantoprazole 80mg bolus followed by 8mg/h	Comments: Reduction of bleeding recurrences in pantoprazole group as compared with the group of H2-antagonists, but this effect was confined only in patients with active bleeding due to gastric ulcers.			
	Ranitidine 50mg bolus followed by 13.5mg/h				

* P < 0.05

Unlike studies from the Eastern countries, European investigations had not confirmed these spectacular results although a beneficial effect was observed. The first study by Daneshmend et al. [21], which included patients with all causes of acute upper gastrointestinal bleeding randomised before endoscopy, showed that omeprazole administration did not improve the clinical outcome of patients, although patients given intravenous omeprazole had less often spots of recent or active bleeding at endoscopy than those who received placebo. Reduction in the percentage of patients with high risk spots of bleeding at endoscopy was also observed in a recent study, where patients were started high doses of omeprazole from admission before endoscopy [25]. Probably the administration of these drugs since admission to the hospital could improve the endoscopic image and the possibility of endoscopic haemostasis. Reduction in rebleeding was also observed in another study from Europe in patients with peptic ulcer bleeding, but in this study patients who received omeprazole had higher mortality [23].

## Endoscopic hemostasis

Early endoscopy (within 24 hours of admission), although does not reduce mortality of patients with acute upper digestive bleeding, is necessary because it helps significantly to establish the cause and site of bleeding as well as spots of active or recent bleeding that are directly related to rebleeding probability [9]. So we can better address these patients (early discharge vs. close intensive monitoring – aggressive treatment).

The introduction of endoscopic hemostasis (endoscopic injection, thermal coagulation, placement of clips or their combination) during the last decades has improved the clinical outcome especially for patients with high-risk stigmata, decreasing the rebleeding rate, blood transfusions requirements, time of hospitalization of patients, the need for urgent surgical haemostasis and probably the fatality rate [9]. Also endoscopic hemostasis is the treatment of choice for lesions not related to acid like angiodysplasias, which are observed with increasing frequency recently.

## Proton pump inhibitors monotherapy or combined with endoscopic haemostasis?

Although proton pump inhibitors administration has beneficial effects in patients with non-variceal upper gastrointestinal bleeding, existing data are insufficient to demonstrate its value as monotherapy (without endoscopic hemostasis) in these patients as well as the ideal regimen of administration [26]. Endoscopic haemostasis is more effective method than any other therapeutic intervention both in patients with active bleeding and in patients with recent bleeding spots (non-bleeding visible vessel or adherent clot).

In a study by Sung et al [27], a total of 156 patients with bleeding from peptic ulcer and non-bleeding visible vessel or adherent clot at endoscopy were studied. All patients initially received intravenous bolus 80 mg omeprazole and then 8 mg/hr continuously for 72 hours, while the patients were divided into two groups. Patients in the first group received endoscopic hemostasis by a combination of adrenaline injection and heatprobe, whilst in the second group no endoscopic therapy was administered. A significant reduction in rebleeding was observed in patients submitted to endoscopic therapy (1.1% vs. 11.6%) indicating that the combination treatment is significantly superior to medication only, which in this case was high-dose intravenous omeprazole continuously.

## Endoscopic hemostasis monotherapy or combined with proton pump inhibitors?

Although endoscopic hemostasis is effective in more than 90% of bleeding cases of non-variceal upper gastrointestinal bleeding, rebleeding occurs in 15-20% of patients after endoscopic hemostasis. In patients at high risk for rebleeding, a second endoscopy 18-24 hours after initial endoscopic treatment for a possible additional therapy does not appear to be effective because the majority of patients do not relapse after successful endoscopic hemostasis. It could however be beneficial in selected cases where hemostasis at first endoscopy was difficult and insecure [9]. On the other hand, a second attempt of endoscopic hemostasis, on rebleeding seems to reduce the rate of surgical hemostasis and complications without increasing mortality in these patients.

Several studies have investigated the possibility of cumulative effect of proton pump inhibitors administration before or after endoscopic hemostasis to prevent recurrence of bleeding. Although there is no consensus, administration of proton pump inhibitors given as a continuous high dose intravenous infusion after endoscopic treatment appears to reduce relapses of bleeding although no study has shown reduced need for emergency surgery or mortality in treated patients (Table 2) [28-37]. Despite these results, meta analysis of 24 studies revealed that the addition of proton pump inhibitors to endoscopic hemostasis, in various regimens, reduces the need for emergency surgery [38]. Also in a recent large study [37], esomeprazole given as a continuous high – dose intravenous infusion after successful hemostasis in peptic ulcer bleeding patients was highly efficacious in reducing rebleeding. In this study, a trend towards reduced need for surgical hemostasis was observed also. These data indicate that, although the benefits from adding proton pumps inhibitors after successful endoscopic hemostasis are not striking, there is a significant cumulative effect indeed.

**Table 2:** Effect of PPIs administration at diverse doses in clinical outcome of patients with bleeding peptic ulcer after endoscopic hemostasis

Studies (Ref. No)		Ν	Rebleeding	Surgery	Mortality
**Javid et al 2001 **[28] Endoscopic injection of adrenaline and polidocanol (India)	Omeprazole orally 40 mg x 2	82	7%	2.4%	1.2%
	Placebo	84	21% *	8.3%	2.4%
**Lau et al. 2000 **[29] Endoscopic injection of adrenaline/thermal coagulation (Hong Kong)	Omeprazole 80mg bolus followed by 8 mg/h	120	6.7%	2.5%	4.2%
	placebo	120	22.5% *	4.2%	10%
**Jensen et al. 2006 **[30] Endoscopic injection of adrenaline/thermal coagulation	Pantoprazole 80mg bolus followed by 8 mg/h IV	72	6.9%	3.5%	4.2%
	Ranitidine 50mg followed by 6.25 mg/h IV	77	14.3% *	4.2%	3.9%
**Ali Zargar et al. 2006 **[31] Endoscopic injection of adrenaline/thermal coagulation (India)	pantoprazole 80mg bolus followed by 8 mg/h	102	7.8%	2.9%	2%
	placebo	101	19.8% *	7.9%	4%
**Udd et al. 2001 **[32] Endoscopic injection of adrenaline/thermal coagulation	Omeprazole 80mg bolus followed by 8 mg/h	73	11.6%	7.2%	5.5%
	Omeprazole 20 mg IV	69	8.2%	4.2%	2.9%
**Kaviani et al. 2003 **[33] Endoscopic injection of adrenaline (Iran)	Omeprazole 20 mg x 4	80	8.5%	1	0
	placebo	80	33.3% *	1	1
**Lin et al 2006 **[34] Endoscopic injection of adrenaline (Taiwan)	Omeprazole 40 mg x 4 IV	67	9%	0%	0%
	omeprazole 40 mg x 2 IV	66	21.2%	0%	1.5%
	Cimetidine 400 mg x 2 IV	67	32.8% *	4.5%	4.4%
**Lin et al. 1998 **[35] Electro- or thermal coagulation	Οmeprazole 40mg bolus followed by 160 mg/day	50	4%	0	0
	Cimetidine 300mg bolus followed by 12000 mg IV /day	50	24% *	0	2
**Ηsu et al. 2004 **[36] Ulcers with stigmata of active or recent bleeding after endoscopic injection	Pantoprazole 40mg bolus followed by 40 mg χ 2	52	3.8%	0	1.9%
	Ranitidine 50mg bolus followed by 50 mg χ 4	50	16% *	2%	2%
**Sung JY et al. 2008 **[37] Ulcers with stigmata of active or recent bleeding after endoscopic injection	Esomeprazole 40 mg bolus followed by 8 mg/h x 3days	375	4.8%*	2.7%**	0.8%
	placebo	389	10.4%	5.4%	2.1%

*P<0.05 and **P=0.059

The majority of studies compared high dose proton pump inhibitors to placebo in patients with peptic ulcer bleeding. There is scarcity of data regarding standard dose of proton pump inhibitors in Western populations although in Eastern populations lower doses are usually sufficient. A recent study showed that there was no difference in rebleeding of patients with peptic ulcer bleeding after successful endoscopic hemostasis treated with high dose omeprazole in comparison to those treated with standard dose [32].

## Conclusions

Endoscopic hemostasis is more effective than any other therapeutic intervention in the treatment of patients with non-variceal upper gastrointestinal bleeding. There are no data on the use of other drugs except proton pump inhibitors in the treatment of patients with acute upper non-variceal bleeding.

Proton pump inhibitors should be started immediately on admission to the hospital and endoscopic examination and probable hemostasis should be performed within 24 hours. Although there is no documentation of the greater effectiveness of high doses of proton pump inhibitors, most studies have compared high doses with placebo, this scheme should be used initially at least in patients with evidence of ongoing and/or severe bleeding and modified thereafter based on the results of endoscopy. In patients with high risk for rebleeding spots, proton pump inhibitors should be continued at high dose for 3 days, while in the rest patients, proton pump inhibitors should be reduced to standard intravenous or even oral doses after endoscopic examination.
